# The development and validation of a genotyping-by-target sequencing chip for fungal population genetic analysis

**DOI:** 10.1007/s44154-025-00281-2

**Published:** 2026-01-19

**Authors:** Haohao Yan, Zhe Ma, Qiang Yao, Shiqin Cao, Qiuzhen Jia, Jiaqi Li, Jie Zhao, Weiyi Yan, Juhong Ma, Wen Chen, Bo Zhang, Xuezhen Ma, Xiaojie Wang, Dejun Han, Zhensheng Kang, Lili Huang, Qingdong Zeng

**Affiliations:** 1 State Key Laboratory for Crop Stress Resistance and High-Efficiency Production, College of Plant Protection, Northwest A&F University, Yangling, Shaanxi 712100 China; 2 State Key Laboratory for Crop Stress Resistance and High-Efficiency Production, College of Agronomy, Northwest A&F University, Yangling, Shaanxi 712100 China; 3 Key Laboratory of Agricultural Integrated Pest Management, Qinghai Province, Academy of Agriculture and Forestry Science, Qinghai University, Xining, Qinghai 810016 China; 4 Plant Protection Institute, Gansu Academy of Agricultural Sciences, Lanzhou, Gansu 730070 China; 5 Plant Protection Institute, Guizhou Academy of Agricultural Sciences, Guiyang, Guizhou 550006 China

**Keywords:** GBTS, *Puccinia striiformis* f. sp. *tritici*, Population genetics, Gene flow, Trajectory tracking

## Abstract

Advanced genotyping technologies for understanding the genetic intricacies of fungal pathogens have broad applications in crop protection. Here, we introduce a novel genotyping-by-target sequencing (GBTS) chip, a versatile tool designed for comprehensive genetic analysis of fungal populations. This technology overcomes key limitations of traditional molecular marker-based approaches by providing a more efficient, economic, and streamlined solution while bypassing the need for labor-intensive pathogen culturing. We demonstrate its utility by applying it to profile *Puccinia* *striiformis* f. sp. *tritici* (*Pst*), the causal agent of wheat stripe rust. Our analysis involved 225 infected leaves collected from wheat fields in the northwest oversummering region for *Pst* in China. We delineated three genetic groups and revealed frequent gene flow, with closer connectivity between Qinghai and Gansu than either province with Ningxia, a pattern consistent with wind trajectory models. These findings illustrate a highly connected regional epidemic system and highlight the value of the GBTS chip for genomic epidemiology. The methodology established here provides a scalable framework for population genetic studies in other fungal pathogens, promising to enhance disease monitoring and management across agricultural systems.

**Supplementary Information:**

The online version contains supplementary material available at 10.1007/s44154-025-00281-2.

## Introduction

Wheat, supplying one-fifth of the world's food calories and protein, is essential for food security (Tilman et al. 2011). However, it is constrained by pathogens and pests, that cause an estimated global yield loss of 21.5%, ranging from 10.1 to 28.1% (Savary et al. 2019). Among these threats, stripe rust caused by the airborne fungal pathogen *Puccinia striiformis* f. sp. *tritici* (*Pst*) is an ongoing global threat that affects wheat at all growth stages (Wellings 2011). This disease has caused eight nationwide epidemics in China over eight decades. Its seriousness lies in its influence on inter-regional pathogen spread, diverse geographic and meteorological conditions, and airborne dispersal (Zeng et al. 2022). The disease cycle involves oversummering between regular crop seasons on wheat grown at higher altitudes or on alternative hosts, overwintering on autumn-sown wheat, and rapid spring spread, predominantly in cool, humid environments (Li and Zeng 2002; Chen and Kang 2017). Deploying and diversifying resistance in wheat cultivars is key to controlling stripe rust, but new pathogen races can arise and quickly overcome resistance genes, leading to "boom and bust" disease cycles (De Vallavieille-Pope et al. 2012). Understanding the population dynamics and migration of *Pst*, especially over long distances, is essential for effective disease management and targeted cultivar deployment (Perrings et al. 2002; Brown 2015). However, advancing our knowledge in this field has been consistently hampered by limitations inherent to available genotyping methodologies.

Consequently, population genetic studies of *Pst* have relied on a succession of molecular markers, each with significant constraints for large-scale epidemiological research. DNA marker systems like Restriction Fragment Length Polymorphism (RFLP), Random Amplified Polymorphic DNA (RAPD), Amplified Fragment Length Polymorphism (AFLP), Simple Sequence Repeats (SSR), and Single Nucleotide Polymorphism (SNP) were applied in studying the population structure of *Pst* (Chen 2005; Hovmøller et al. 2011). RFLP, despite providing excellent replicability and revealing significant geographical variation, was limited in application since it was time-consuming, costly, and required radioactive materials (Saiki et al. 1985; Shan et al. 1998). RAPD, though simple and user-friendly, had low replicability within and between laboratories (Williams et al. 1990; Chen et al. 1993). AFLP was efficient and cost-effective but only identified dominant markers (Vos et al. 1995). SSRs, extensively employed for investigating population structure and migration, highlighted the significance of pathogen variation in the Himalayan region but were inappropriate for large-scale genotyping due to low marker density (Enjalbert et al. 2002; Ali et al. 2014). SNPs, facilitated by next-generation sequencing, enhanced understanding of genetic diversity and migration of *Pst* but high-throughput genotyping can be costly (Mardis 2008; Xia et al. 2016a; Chen and Kang 2017; Ding et al. 2021; Li et al. 2023). Additionally, as an obligate biotrophic fungus, *Pst* must be cultured on living wheat plants for growth and survival. Conventional research methods involve labor-intensive procedures, including isolate purification and reproduction, which also elevates the risk of cross-contamination among samples. Despite its small size of under 100 Mb, the *Pst* genome remains largely uncharacterized due to challenges imposed by dikaryotic urediniospores and obligate parasitism. Direct DNA extraction from uredinia and transcriptomic sequencing of infected wheat leaves were employed to address these issues (Ali et al. 2011; Hubbard et al. 2015), but the methods were costly and primarily provided host-derived data, limiting our understanding of the pathogen.

To overcome these limitations, we turned to genotyping-by-target sequencing (GBTS), a targeted sequencing strategy that offers a tailored solution for pathogen population genetics. Recent multiple target-enrichment strategies have advantages in high throughput, low time requirements, and cost effectiveness (Mamanova et al. 2010). The GBTS platform combines the advantages of high-throughput sequencing with the cost-effectiveness and focus of targeted capture, either by multiplexing PCR (GenoPlexs) or probe hybridization (GenoBaits) (Guo et al. 2019). This simplified genome sequencing approach significantly minimizes the amount of DNA sequencing and simplifies subsequent biological information analysis and data processing (Xu et al. 2020). The repeatability of the approach ensures consistent genotype acquisition regardless of laboratory conditions and sequencing platforms, and fosters data comparability, integration, and sharing (Xiang et al. 2023). GBTS can accurately capture genomic regions of any length or position, encompassing diverse elements such as exon and genome fragments. As this capability extends to simultaneous detection of genetic variations, including SNPs, SSRs, InDels, fusion genes, and methylation sites (Guo et al. 2022), it is an ideal approach for population genetic characterization of plant pathogens.

GBTS was developed and widely applied across more than 20 crops, vegetables, animals and microorganisms, but has not been developed and applied in fungal related research (Guo et al. 2019; Xu et al. 2020; Shen et al. 2021; Guan et al. 2023). In a previous study by our laboratory, GBTS exhibited stability, reliability, flexibility, and cost-effectiveness (Xiang et al. 2023). The replicability of the approach ensures consistent genotype acquisition regardless of laboratory conditions and sequencing platform, fostering data comparability, integration, and sharing (Xiang et al. 2023). In the present study, we developed a GBTS-based genomic chip for *Pst* based on genome re-sequencing data. Analysis of a collection of wheat stripe rust samples from the northwest oversummering region in China was undertaken to validate the chip and demonstrated genetic relationships among the pathogen populations. The inclusion of a significantly greater number of markers than in previous studies increased the resolution of genetic analysis using the chip, making it a valuable tool for understanding genetic diversity and migration of *Pst* both within and between regions. Ultimately, the *Pst* 20 K GBTS chip will contribute to the development of comprehensive strategies for control of wheat stripe rust and contribute to ongoing food security. This advancement is not only instrumental for deciphering the genetic diversity and migration patterns of *Pst* across regions but also holds broad applicability for population studies of other pathogenic fungi.

## Results

### Comprehensive quality control and validation of SNP variants in *Pst* 20 K GBTS chip analysis

**Fig. 1:**
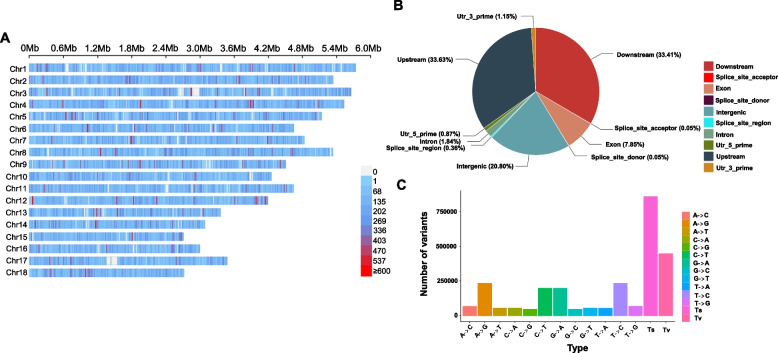
Single nucleotide polymorphism (SNP) data from the genotyping-by-target sequencing (GBTS) chip. **A** Distribution of SNP markers serving as markers within 10 kb windows across 18 *Puccinia striiformis* f. sp. *tritici* chromosomes. Marker density is represented by color. The number of each bar represents the SNP number of 10 kb window size. **B** SNP marker annotations. Exon, Expressed region; Utr_5_prime, 5’ untranslated region; Utr_3_prime, 3’ untranslated region. **C** Distribution of SNP types. A-** > **C, Nucleotide base A mutates to C; A-** > **G, Nucleotide base A mutates to G; A-** > **T, Nucleotide base A mutates to T; C-** > **A, Nucleotide base C mutates to A; C-** > **G, Nucleotide base C mutates to G; C-** > **T, Nucleotide base C mutates to T; G-** > **A, Nucleotide base G mutates to A; G-** > **C, Nucleotide base G mutates to C; G-** > **T, Nucleotide base G mutates to T; T-** > **A, Nucleotide base T mutates to A; T-** > **C, Nucleotide base T mutates to C; T-** > **G, Nucleotide base T mutates to G; Ts, Transitions; Tv, Transversions

Sequencing of the 225 *Pst* samples with the *Pst* 20 K GBTS chip yielded 361.9 Gb of raw data. After quality filtering, 323.9 Gb of high-quality clean reads were retained, yielding an effective rate (ratio of filtered clean data to raw data) of 80.78–94.79%. This high rate indicated successful removal of low-quality sequences and contaminants, ensuring a reliable foundation for variant identification. The mapping rate to the primary contig of the *Pst* reference genome (Australian race 134E16A + 17 + 33 +) ranged from 45.88% to 95.89%, with a high mean of 85.12%. This demonstrates the exceptional specificity of our GBTS chip design, confirming that the majority of captured sequences originated from the target *Pst* genome, which is crucial for accurate SNP calling (Table S1). After processing, there were 1,293,150 SNPs in the raw VCF output with a maximum gap of 99,512 bp; only 40 regions were larger than 20,000 bp (Fig. 1A; Table S2). Among polymorphic regions examined, the highest proportion of variants (up to 33.63%) was in upstream regions, followed closely by downstream regions (33.41%). SNPs in intergenic and exon regions accounted for 20.80% and 7.85%, respectively; other regions exhibited fewer variants (Figs. 1B and S1A). With respect to mutation types, transitions (Ts) were nearly twice as frequent as transversions (TVs) (Fig. 1C). SNPs in the CDS region were annotated and statistically analyzed to measure genetic diversity and high-resolution differences between isolates using the high-density molecular markers. Among variants, non-synonymous single nucleotide variants (SNVs) and synonymous SNVs represented the highest proportion, whereas stop gain and stop loss variants constituted the lowest proportion (Fig. S1B).

To ensure the accuracy of further analyses, loci were removed if they had more than two alleles, if the minimum depth of total or alternative allele read coverage was below 3, or if > 25% of the isolates had missing data for the region of interest. A total of 105,542 SNPs passed this quality control (QC) step. For population structure, phylogenetic, and other genetic analyses, loci with missing values or MAF < 0.05 were removed, yielding 19,139 SNPs (https://github.com/zengqd/PopulationGenetics/blob/main/Fungi/PstGTBSChip/TargetGenesAndSNP.xlsx). In addition to these SNP loci, 90 published SSR markers were genotyped with the chip (Table S3), enabling comparisons of the data to previous findings. Having validated the approach and tested markers, the new tool was named the *Pst* 20 K GBTS chip.

### Achievrment of high-throughput genotyping with multifaceted advantages using the GenoBaits GBTS chip

Our GenoBaits GBTS utilizes capture-in-solution (liquid chip) to selectively target genomic regions of interest for high-throughput sequencing. This method provides exceptional resolution, cost-effectiveness, and adaptability, enabling the customization of probes to match specific research objectives. By concentrating solely on the targeted regions, GenoBaits simplifies data analysis, minimizing computational demands and facilitating swift identification of genetic variations. The per-sample expenses for the *Pst* 20 K GBTS chip was ~ 15 US dollars, encompassing DNA extraction ($0.8), library construction ($5), probe hybridization ($4), sequencing ($3), bioinformatics analysis ($0.5), labor ($1.5), and depreciation ($0.2). The cost per sample for a SSR marker was ~ 1 US dollar (Sangon, Shanghai). The cost per sample per KASP marker is 0.1 US dollar (Xiang et al. 2023). Notably, other sequencing methods are costly and are unsuitable for large-scale sample sequencing in population genetics study (Table S4).

The GBTS technology achieves highly reproducible locus coverage through its specific probe design. Compared to GBS and RAD-seq, which depend on restriction enzymes and are susceptible to inconsistent coverage and allele dropout due to enzyme site variation, GBTS provides uniform and reliable genotyping. Compared to whole-genome sequencing, GBTS avoids generating masses of redundant data while providing sufficient marker density for population genetics, significantly streamlining downstream analysis. A decisive advantage of our approach is its compatibility with direct genotyping using field-infected leaves. This bypasses the need for the lengthy urediniospore amplification cycle—up to three months for traditional methods like SSR and KASP—which involves a degree of contamination risk. Consequently, the research timeline is shortened from months to weeks, while eliminating the risk of admixture during culturing. This offers a more authentic snapshot of the pathogen's population structure in the field. Compared to field transcriptomics—another NGS-based method—GBTS is not constrained by RNA instability, complex sampling requirements, or the inherent difficulty of translating expression data into genotype information. Together, these attributes establish GBTS as a methodology that balances cost, efficiency, reliability, and practicality for large-scale population genetic studies in obligate biotrophs, as supported by the high-throughput performance of the *Pst* 20 K GBTS chip (Table S4).

### Dynamics of *Pst* migration in the northwest oversummering region

**Fig. 2:**
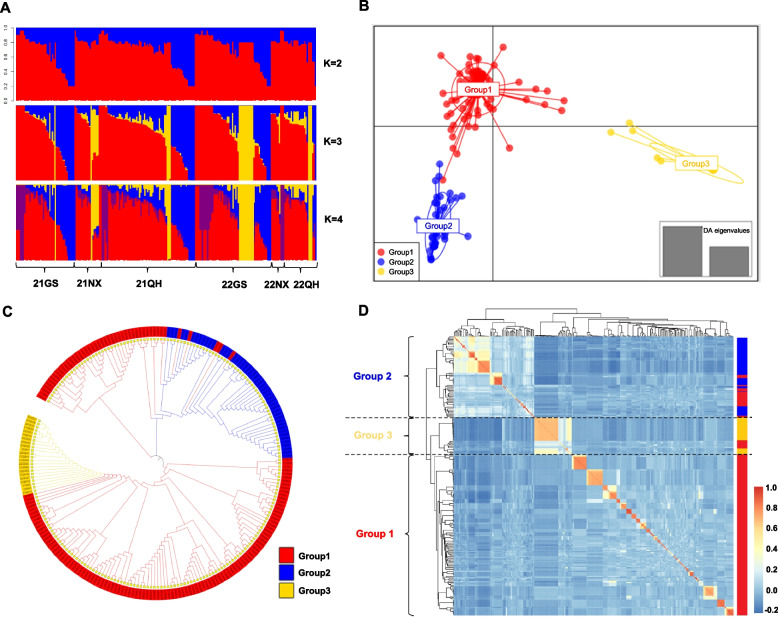
Population genetic structure of 225 *Puccinia striiformis* f. sp. *tritici* (*Pst*) isolates collected from the northwest oversummering region of China during 2021 and 2022. **A** Assignment of isolates to genotype clusters using STRUCTURE. Each line represents an isolate and was partitioned into one of the K clusters. 21GS, 2021 Gansu; 21NX, 2021 Ningxia; 21QH, 2021 Qinghai; 22GS, 2022 Gansu; 22NX, 2022 Ningxia; 22QH, 2022 Qinghai. **B** Discriminant analysis of principal components (DAPC) showing clear separation of three groups. **C** Neighbor-joining phylogenetic analysis of the 225 *Pst* isolates using single-nucleotide polymorphisms (SNPs) filtered by linkage disequilibrium (LD). **D** Kinship matrix based on simple matching of genetic similarities

To demonstrate the application of the GBTS chip in resolving key epidemiological questions, we employed it to study the migration dynamics of *Pst* in the northwest oversummering region. The high-density SNP data generated by the chip enabled a detailed investigation into the population structure and genetic diversity of the pathogen. The Evanno method (implemented in STRUCTURE) identified K = 3 as the optimal number of genetic clusters (Fig. S2A) that also aligned with the three provinces, providing a biologically relevant framework for analysis. At K = 2, the isolates from Qinghai, Gansu, and Ningxia divided into two genetic groups distributed across all six populations (Fig. 2B). At the optimal K = 3, the population was subdivided into three distinct genetic groups. Group 1 (red, containing 153 isolates) was the dominant cluster, followed by Group 2 (blue, 48 isolates), and Group 3 (yellow, 24 isolates) (Table S1). The population structure was largely consistent between 2021 and 2022 across the three provinces. A notable finding was that the 22GS population from the Longdong region showed genetic divergence from the other 22GS samples in 2022 but closely resembled that of the 21NX population from Ningxia, suggesting a close genetic relationship and potential migration between these groups. While higher K-values (up to K = 9) were evaluated, the substructures observed, such as at K = 5, did not receive strong statistical support from the ΔK method. Nevertheless, the consistent distinctness of Group 3 (yellow) across all K-values underscores its unique and stable genetic identity within the regional population (Fig. S2B). Furthermore, STRUCTURE analysis revealed the presence of admixed individuals across all six populations, as defined by an ancestry proportion between 10 and 90% from any genetic group (see Methods). These hybrids provide direct genetic evidence of ongoing gene flow and genetic exchange. For instance, within the 21GS population, 28 isolates (including 21GS16, 21GS20, and 21GS22) were identified as admixtures of Group 1 and Group 2. More complex ancestry was observed in isolates like 21GS1 and 21GS8, which exhibited genetic contributions from all three groups, underscoring the complexity of genetic interactions in the region.

DAPC analysis reinforced the findings of STRUCTURE, categorizing all genotypes into three groups (Fig. 2B). Group 1 represented a significant portion of all six populations and emerged as the primary component. Group 2 was most abundant in the 21GS, 22GS, and 21QH populations. Group 3 was mainly found in the 21NX, 22QH, and 22GS populations, but was also present in small proportions in the 21GS and 21QH populations. In 2022, the Ningxia population displayed a notable shift; it retained genotypes from Group 1 in 2021 and lost the Group 2 and Group 3 genotypes, a significantly altered genetic landscape (Fig. S3).

Phylogenetic analysis validated the divisions between populations, yielding three lineages (Groups 1, 2, and 3) (Fig. 2C). This analysis highlighted the close genetic relationship between isolates from Groups 1 and 2, underscoring their separation from those in Group 3, consistent with the DAPC results. Despite the presence of just three primary clusters across multiple analyses, each group exhibited internal subdivisions, indicating smaller-scale genetic exchanges among isolates within each region. Finally, genetic relationships were visualized with a kinship heatmap, which corroborated division of the 225 isolates into the three groups (Fig. 2D). In summary, STRUCTURE, DAPC, phylogenetic, and kinship analyses illustrated the dynamic nature of pathogen exchange throughout the northwest oversummering region and supported the existence of three main genotypic groups.

### Genetic diversity in *Pst* populations

To first benchmark the resolution and discriminatory power of the GBTS chip, we analyzed the genetic diversity across all 225 sampes. The chip successfully identified a unique multi-locus genotype (MLG) for each sample, demonstrating its high resolution at the strain level. The overall populations were stratified into distinct MLGs, corresponding to collection sites in each year: 21GS (44 MLGs), 21NX (19), 21QH (71), 22GS (57), 22NX (7), and 22QH (27). After post-clonal sample size correction, the 22NX population exhibited the lowest expected MLG (eMLG) count at 7, whereas the other five populations all had eMLGs of 10. Genetic diversity analyses underscored the remarkable polymorphism within SNP sites (ranging from 96.75–100.00%), affirming the comprehensive polymorphic coverage of the chip. All six populations displayed high observed allele numbers, averaging 1.976. Remarkably, the 21GS, 21QH, and 22GS populations all had the highest observed number of alleles (*Na* = 2.00), whereas the 22NX population had the lowest (*Na* = 1.868). Similar patterns were evident for effective allele numbers, with 21NX exhibiting the highest value (*Ne* = 1.665) and 22NX having the lowest (*Ne* = 1.547).

The Shannon diversity index (*I*) showcased variation between the populations; diversity was highest in the 21NX population (*I* = 0.556), followed by the 21QH population (*I* = 0.542) and the 22NX population (*I* = 0.466). *Ho* surpassed *He* in all populations, with the 21NX population showing the highest *Ho* (0.522) and *He* (0.378). Heterozygosity was lowest in the 22NX population (*Ho* = 0.445, *He* = 0.313) (Table 1). Overall, these results demonstrated high genetic diversity in the 21NX population and low genetic diversity in the 22NX population (post-winter), consistent with the DAPC results.

**Table 1:** Genetic diversity among six *Puccinia striiformis* f. sp. *tritici* populations

Pop	*N*	MLG	eMLG	*Na*	*Ne*	*I*	*Ho*	*He*	*P* (%)
21GS	44	44	10	2.000	1.628	0.540	0.427	0.363	100.00
21NX	19	19	10	1.994	1.665	0.556	0.522	0.378	99.41
21QH	71	71	10	2.000	1.629	0.542	0.431	0.364	100.00
22GS	57	57	10	2.000	1.614	0.533	0.417	0.357	99.98
22NX	7	7	7	1.868	1.547	0.466	0.445	0.313	96.75
22QH	27	27	10	1.997	1.612	0.529	0.432	0.355	99.68
**Total**	**225**	**225**	**10**	**1.976**	**1.616**	**0.528**	**0.446**	**0.355**	**99.30**

Pop, population name; *N*, number of isolates; MLG, multi-locus genotype; eMLG, expected MLG count; *Na*, observed number of alleles; *Ne*, effective number of alleles; *I*, Shannon index; *Ho*, observed heterozygosity; *He*, expected heterozygosity; *P*, percentage of polymorphic loci; 21GS, 2021 Gansu; 21NX, 2021 Ningxia; 21QH, 2021 Qinghai; 22GS, 2022 Gansu; 22NX, 2022 Ningxia; 22QH, 2022 Qinghai

### High levels of gene flow within *Pst* populations in northwest China

**Fig. 3:**
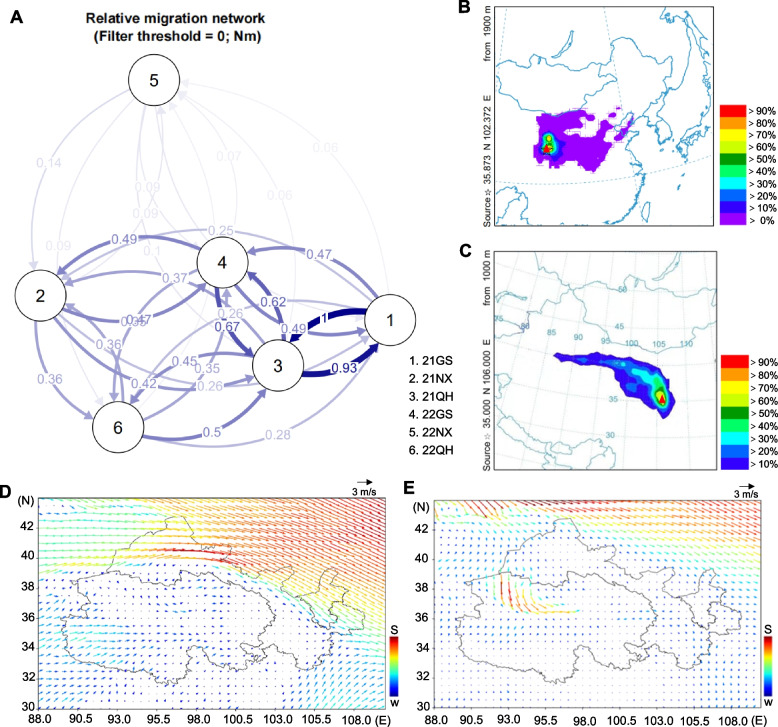
Gene flow (*Nm*) patterns within epidemiological sub-regions of northwest oversummering region and inference of the direction of *Puccinia striiformis* f. sp. *tritici* spread based on meteorological data. **A** Migration network showing gene flow (*Nm*) patterns within epidemic sub-regions. Line thickness indicates *Nm* intensity. 21GS, 2021 Gansu; 21NX, 2021 Ningxia; 21QH, 2021 Qinghai; 22GS, 2022 Gansu; 22NX, 2022 Ningxia; 22QH, 2022 Qinghai. 0.0 < *Nm* ≤ 0.25, low level of gene flow; 0.25 < *Nm* < 0.75, moderate level of gene flow; 0.75 ≤ *Nm* ≤ 1.0, high level of gene flow. **B** Inference of the spore movement trajectory in September 2021. Salar Autonomous County in Qinghai province was designated the central point (red triangle). **C** Inference of the spore movement trajectory in May 2022. Maiji District (red triangle), Gansu province was designated the central point. **D** Upper air wind field map from September–December 2021. S, strong; W, weak. Arrow color corresponds to wind intensity. N, North; E, East. **E** Upper air wind field map from April–July 2022. N, North; E, East

We next leveraged the genome-wide data to test the chip sensitivity in quantifying population connectivity. *Fst* values for the six populations ranged from 0.005–0.053, AMOVA indicated that the differences between samples and sampling times were statistically significant (*p* < 0.05) (Table S5, Table S6). D values ranged from 0.006–0.061, further demonstrating a lack of genetic divergence among the populations. To distinguish genuine gene flow from signals of incomplete lineage sorting (ILS), we performed *D*-statistics (ABBA-BABA tests). The results significantly rejected the null hypothesis of no gene flow for multiple population pairs (|Z-score|> 3, *P* < 0.05. Fig. S4, Table S7), corroborating the variable but widespread migration indicated by the *Nm* values (ranging from 0.06 to 1.0; Fig. 3A). This independent validation confirmed that the observed genetic connectivity was driven by actual migration events rather than ancestral polymorphism. Robust gene exchange in 2021 was noted across all three populations, particularly between the 21QH and 21GS populations. Conversely, the genetic relationships between the Ningxia and Gansu populations remained relatively weak in 2021, despite the geographical proximity of these locations. This interesting pattern indicated a unique link between the Qinghai and Ningxia populations, consistent with the STRUCTURE results.

Although substantial genetic exchange occurred among the populations in 2022, these interactions were significantly diminished compared to 2021. There was clear proximity between the 22QH and 22GS populations, whereas both populations had more distant relationships with the 22NX population. Notably, five of the populations (except 22NX) exhibited robust gene flow. The affinity between the 21QH and 22GS populations surpassed the affinity between the 21GS and 22QH populations, suggesting a significant contribution from 21QH to the 22GS population in the oversummering period (Fig. 3A). In summary, during 2021 and 2022, populations in the northwest China epidemic region underwent frequent gene exchange, with the Qinghai and Gansu populations demonstrating a closer genetic relationship than either with the Ningxia population. These results provide valuable insights into the intricate dynamics of *Pst* dispersal.

### Wind movement and trajectory models that might affect *Pst* migration

To move beyond genetic patterns and towards an integrated understanding of dispersal, we combined our genetic findings with atmospheric data. The general trend of high-altitude wind movement in the northwest oversummering region from September to December 2021 was west to east (Fig. 3D). Theoretically, this would allow urediniospores to move from Qinghai to Gansu and to Ningxia Autonomous Region. A HYSPLIT-4 trajectory model showed movement from a central source (Salar Autonomous County, Qinghai) to the wheat growing areas of Gansu and Ningxia (probability > 10%). There was also a high probability that spores from the central source would spread by airflow to the major wheat growing areas of Henan and Hubei further east (Fig. 3B). From April to July 2022, there were two general wind trends in western and central Qinghai: one from Xinjiang to Qinghai and the other from Guangyuan city in Sichuan province to eastern Qinghai via Gansu (Fig. 3E). A trajectory model for this period showed a likely spread from a central source represented by Tianshui city, Gansu from east to west, covering part of Ningxia and the eastern wheat area of Qinghai (probability > 30%) (Fig. 3C). Thus, the upper air wind data during these two periods were consistent with the results of the HYSPLIT-4 trajectory models.

### Temporal dynamics of stripe rust epidemics in northwestern China

To unravel the epidemic pattern of *Pst* in the northwest oversummering region of China, field surveys were conducted for the three sampling regions in the 2021 and 2022 cropping seasons. Observations made from August to December 2021 revealed that infection type (IT), disease severity (DS), and disease prevalence (DP) in Qinghai Province were notably higher compared to Gansu and Ningxia (Table S8). Furthermore, following the harvest of wheat in Ningxia and Gansu in August, infections persisted in volunteer winter wheat, creating a "green bridge". Similarly, in Qinghai, besides volunteer wheat, a significant presence of spring-sown wheat also contributed to the carryover of inoculum. Indeed, the incidence of fields with infected volunteer wheat seedlings in Qinghai surpassed that of Gansu and Ningxia. Consequently, winter wheat planted in Qinghai and Gansu in October exhibited infection in November 2021, whereas the disease was not recorded in Ningxia until December, following the pattern observed in Qinghai and Gansu. In 2022, the temporal progression of the wheat stripe rust displayed a dynamic pattern; it was first observed in Longnan in mid-May, and then the Tianshui and Longdong areas in late May, followed by Linxia Hui Autonomous Prefecture in mid-June. Stripe rust was not observed in Qinghai or Ningxia until early June (Table S8). These findings underscore the nuanced interplay between environmental factors and pathogen dynamics, illustrating the complex epidemiological factors contributing to stripe rust epidemiology in this region.

## Discussion

Although genome-wide sequencing offers comprehensive insights into genomic variation current applications in the population genetics and dynamics of wheat stripe rust are limited by high cost, especially for large-scale sample analyses. Furthermore, traditional genotyping methods that rely on PCR markers or next-generation sequencing of *Pst* necessitate multiple cycles of urediniospore increase, requiring approximately three months, with inherent risk of admixture in the laboratory. To fulfill the unmet need for a high-throughput, low-cost *Pst* genotyping solution, we leveraged GBTS technology to develop the present *Pst* 20 K GBTS chip, which includes SNPs, SSRs, and InDels, providing an extensive repertoire of markers accessible through liquid capture. The *Pst* 20 K GBTS chip allows for direct use of diseased leaves collected from the field thus saving time and labor. Overall, the chip is significantly less expensive per sample than PCR-based methods. Compared with whole genome sequencing, it does not increase the difficulty of data processing and does not generate a large amount of redundant data. In summary, the *Pst* 20 K GBTS chip allows identification of a rich spectra of genomic variation in the *Pst* genome while providing high coverage, precision, and efficiency. The analysis pipeline was provided to the MolBreeding Biotechnology Company and is publicly accessible at https://github.com/zengqd/PopulationGenetics/tree/main/Fungi/PstGTBSChip/Step1_1fq2vcf.sh. The pipeline requires only simple genotype data as input and includes filtering and standard genetic analyses, such as DAPC, kinship analysis, and phylogenetic tree construction. This enables broad use of our method by researchers without expertise in bioinformatics.

Our genotypic analysis using the *Pst* 20 K GBTS chip revealed significant genetic connectivity among *Pst* populations in the northern oversummering region of China. Crucially, the widespread distribution of admixed individuals, identified through STRUCTURE analysis, offers a direct genetic footprint of this connectivity. We propose that the emergence of these variants is facilitated by sexual reproduction on the widely distributed alternate host, *Berberis* spp., in this ecologically complex region. This process allows for genetic recombination between genotypes of distinct genetic backgrounds (Groups 1, 2, and 3). Recombination is a critical mechanism for virulence evolution, as it shuffles existing pathogenicity alleles into novel combinations, potentially giving rise to new and more aggressive pathotypes. The cross-regional presence of Group 3, now traceable through admixture patterns, exemplifies how long-distance spore dispersal, coupled with local recombination, can rapidly disseminate and reshuffle genetic traits (Chen et al. 2014; Kong et al. 2014; Yao et al. 2014). Therefore, the identification and monitoring of admixed individuals are critical for forecasting the emergence of virulent races and devising proactive disease management strategies.

Our findings on the high levels of gene flow and genetic connectivity are consistent with and reinforce = migration relationships described in previous studies. Chen and Kang (2017) explicitly classified Gansu and Qinghai as part of the same inoculum source region, noting similar virulence structures and frequent spore exchange. This is further supported by population genetic studies; Zhan et al. (2022) reported strong gene flow between Gansu and Qinghai using SSR and KASP markers, while Zhu et al. (2025) documented significant transmission among Qinghai, Gansu, and Ningxia using SSR analysis. Our study, employing a high-density GBTS chip, not only confirms these robust connections within the northwest oversummering region but also provides finer resolution by identifying the specific genetic groups (e.g., admixed individuals, Group 3) that are actively participating in these migration events.

Extensive field investigations spanning Qinghai and Gansu and Ningxia during 2021 and 2022 yielded insights into the survival of *Pst* in the region. Our in-depth analysis of epidemiology in the northwest oversummering region suggests a departure from previous reports. The Longnan area in Gansu has historically been identified as a pivotal hotspot for generating and maintaining variation in *Pst* virulence, although wheat stripe rust consistently encroaches upon the entire wheat planting area (Chen et al. 2013). Our investigation from June 2021 to August 2022 revealed shifting patterns in epidemic dynamics. In 2021, disease prevalence followed a distinctive order: Qinghai exhibited the highest proportion of fields with stripe rust (84.04%), followed by Gansu (75.34%), and Ningxia (71.43%). The disease levels in Qinghai Province ranged from 5—50%, slightly surpassing Gansu (5—40%) and significantly exceeding Ningxia (1—20%) (Table S8). The higher prevalence and severity rates in Qinghai were likely due to the presence of late-maturing spring wheat and abundant volunteer wheat seedlings favoring *Pst* survival. This contrasts sharply with the declining suitability of fields in Gansu and Ningxia for post-harvest survival of *Pst* due to climatic shifts and changes in agricultural practices (Chen et al. 2013). This marked shift in host dynamics redefines our understanding of *Pst* epidemiology, with significant implications for crop management and resistance strategies.

Our dissection of *Pst* population genetics across the region lays the foundation for strategic crop planning and disease control. At least some of the candidate genes encoding secreted proteins included in the *Pst* 20 K GBTS chip are expected to be the products of avirulence alleles that will be identified in future studies (Xia et al. 2016b). Thus, our study has significant molecular ecological implications, offering novel insights into wheat stripe rust epidemiology, and host–pathogen interactions. Wheat stripe rust epidemiology is a comprehensive study carried out on *Pst* populations worldwide, and such studies must be done on a regular basis to track the pathogen dynamics. The GBTS strategy established here presents a scalable paradigm for population genetic studies in other major fungal pathogens. As the approach is built upon the versatile foundation of next-generation sequencing (NGS) platforms, adapting it to new pathogens primarily requires only the in silico design of specific probes. Custom chips can thus be efficiently developed for *Blumeria graminis* (pathogen of powdery mildew), *Fusarium* species (Fusarium head blight), or *Magnaporthe oryzae* (rice blast), enabling high-resolution tracking of their migration and evolution without the need for prior sub-culturing.

When considering its role in disease early-warning systems, our GBTS approach complements other emerging technologies. For instance, field transcriptomics—pioneered in *P. striiformis* (Hubbard et al. 2015) and recently applied to *P. polysora* (Li et al. 2025)—excels at capturing functional, gene expression data from field samples, providing a real-time snapshot of pathogen activity. However, its utility can be limited by RNA instability and the difficulty of translating transcriptomic reads into stable genotypes for long-term lineage tracking. Moreover, as the vast majority of sequencing reads originate from the host plant, achieving sufficient depth for the pathogen necessitates extensive sequencing, consequently increasing both sequencing and computational analysis costs (Li et al. 2025). In contrast, our GBTS chip generates robust, reproducible genotypic data directly from pathogen DNA, making it uniquely suited for deciphering population structure, inferring migration routes, and monitoring the rise and spread of specific virulent lineages over time. Together, these methodologies can form a more comprehensive early-warning framework.

Our study underscores the role of wind in *Pst* transmission. High-altitude wind mapping and trajectory analyses unveiled important prevailing wind patterns, suggesting potential long-distance dispersal routes, particularly from Xinjiang to the northern oversummering region. Detection of Group 3 genotypes in disparate locations spanning Qinghai, Ningxia, and the Longdong area of Gansu suggested intriguing cross-regional connections. Combined with the wind patterns from September to December 2021, the wind from Xinjiang spread from west to east along the Qilian Mountains to Ningxia and the Longdong area, it is speculated that Group 3 originated from Xinjiang, a pattern that needs to be verified in the future. These findings indicate an urgent need for greater in-depth validation of survival and spread of *Pst* in China. We expect the *Pst* 20 K GBTS chip developed in this work will play an important role in more extensive studies covering a much wider area and involving a greater period of time.

## Materials and methods

### Identification of a genome-wide SNP for chip development

In our previous work, we developed two wheat breeding chips based on genotyping by target sequencing (GBTS) technology. The same wheat variety was repeatedly subjected to chip sequencing to validate reproducibility. The call rate among duplicate samples ranged from 95.34 to 95.76%, and the genotypic consistency ranged from 99.47% to 99.58% (Liu et al. 2025). Furthermore, a validation series confirmed that these chips provide high-throughput, robust, and cost-efficient genotyping data, enabling rapid screening of favorable allelic variants in germplasm resources, parental lines, and breeding populations (Xiang et al. 2023). Building on these successful applications of GBTS-based chip development, we have now extended this platform to fungal population genetics.

**Fig. 4:**
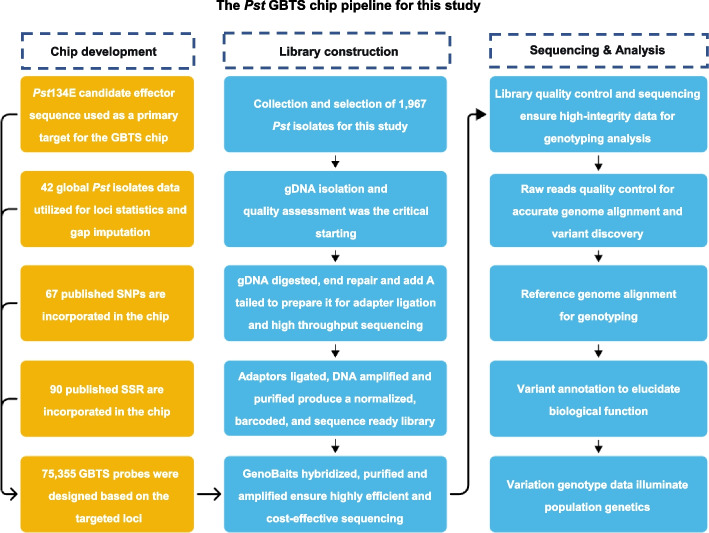
Application of the genotyping-by-target sequencing (GBTS) chip in genotyping *Puccinia striiformis* f. sp*. tritici* isolates. Quality control pipeline: involving the filtering of low-quality raw reads, stringent hard-filtering of initial variants (QD < 2.0, QUAL < 30.0, FS > 200.0, ReadPosRankSum < −20.0), exclusion of low-depth sites (DP < 10), and finally, linkage disequilibrium pruning to obtain a core set of independent SNPs for population genetic analysis

The *Pst* GBTS development pipeline is shown in Fig. 4. The first chromosome-scale phased assembly of an Australian isolate of *Pst* race 134E16A + 17 + 33 + (*Pst*134E36_v1_pri) (Schwessinger et al. 2022) was used as the reference genome in developing a reliable and balanced genotyping chip. Probes were designed from the entire genomic DNA sequence (from start codon to end codon) encoding all predicted effector proteins (https://github.com/zengqd/PopulationGenetics/blob/main/Fungi/ PstGTBSChip/ProbesInformation.xlsx). Polymorphism data from 42 *Pst* isolates collected worldwide were used for basic marker statistics and gap filling. For comparison with previous studies, probes were also designed for 67 SNP loci (Xia et al. 2016a; Meng et al. 2020) and 90 SSR loci (Cheng et al. 2012; Bailey et al. 2015; Luo et al. 2015). Target loci were included if they met the following criteria: SNPs with minor allele frequency (MAF) > 0.1 to exclude rare alleles and enhance the robustness and portability of markers for population analyses; a missing data rate < 30% to ensure reliable genotyping across diverse samples; and heterozygosity < 30% to select for high-confidence, stable genomic positions, minimizing potential artifacts arising from the dikaryotic nature of *Pst* or sample impurities. Finally, 6,813 regions were selected, and 75,355 probes (100 bp for each probe) were designed and synthesized (https://github.com/zengqd/PopulationGenetics/blob/main/Fungi/PstGTBSChip/ProbesInformation.xlsx).

### Field surveys, sampling strategy and collection

Field surveys were carried out during the cropping seasons from June 2021 to August 2022 in Qinghai and Gansu provinces and Ningxia Autonomous Region. These regions experience relatively low temperatures in summer that allow wheat stripe rust to oversummer, while some regions had mild winter climates suitable for overwintering. Therefore, wheat stripe rust can complete the annual cycle in these regions and continue to provide urediniospores for the main wheat production regions (Li and Zeng 2002). To ensure the collection of primary disease samples across various regions within the northwest oversummering area, we followed the methodology outlined in Li and Zeng (Li and Zeng 2002). By considering historical epidemic patterns and monitoring of wheat stripe rust incidence in advance our objective was to gather more representative samples. Throughout each province/autonomous region, three or four wheat fields at least 15 km apart in each selected county were surveyed and sampled. Detailed field data were recorded, including geographical coordinates, altitude, disease prevalence (DP), ecological characteristics, growth stages, disease response infection type (IT), and disease severity (DS). Disease prevalence was quantified as the ratio of diseased leaves to the total number of leaves surveyed in each field. A five-point investigation method was used to investigate each field; five points were randomly selected in each field and 100 leaves were inspected at each point, respectively. The DP of each field was obtained from the total number of infected leaves at five points/500*100%. Additionally, the diseased field frequency (DF) was computed as the number of infected fields/total number of surveyed fields*100% (Li and Zeng 2002). IT, the response of a wheat plant to stripe rust, was recorded according to a 0–9 scale (Line and Qayoum 1992). DS was recorded as the percentage of diseased leaf area to the total leaf area (Chen et al. 2002).

**Fig. 5:**
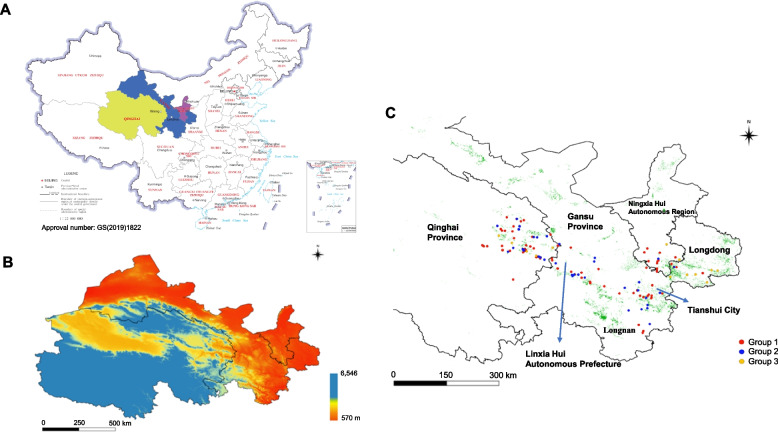
Wheat stripe rust sampling sites. **A** Geographical location of the northwest oversummering region, comprising Qinghai province (olive), Gansu province (blue), and Ningxia Autonomous Region (red). **B** Altitude map for the northwest oversummering region. Colors represent different altitudes. **C** Locations of group members of *Puccinia striiformis* f. sp*. tritici* isolates collected during 2021 and 2022. Wheat cultivation areas in the northwest oversummering region are shown in green shading

Wheat leaves with a single *Pst* uredinium were collected. Three to four leaves with single uredinia were selected at each sampling site from different fields in each county. In total, there were 225 samples; 134 were collected June—December 2021 and 91were collected May- -August 2022. The first group comprised 44 samples (21GS) from 13 counties in Gansu, 71 samples (21QH) from 12 counties in Qinghai, and 19 samples (21NX) from four counties in Ningxia; and the second group included 57 samples (22GS) from 19 counties in Gansu, 27 samples (22QH) from nine counties in Qinghai, and 7 samples (22NX) from two counties in Ningxia. Samples were taken to the laboratory and freeze dried for 2 d, prior to storage at 4 °C in a desiccator with silica gel until further use. The detailed procedure for processing each leaf is described in the supplementary information (Text S1). Detailed sample metadata, including sampling site, altitudes, and coordinates, are shown in Fig. 5 and Table S1.

### DNA extraction and sequencing

Four 6 mm diameter samples were excised from the infected area of each leaf using a punch. DNA was extracted using the CTAB protocol (Saghai-Maroof et al. 1984). The extracted DNA was initially treated with the GenoBaits End Repair Kit and sequencing adaptors and rigorously purified through a series of steps including hybridization with specific baits, purification with washing buffer, and post-PCR amplification prior to genotyping by MolBreeding Biotechnology Co., Ltd. (Shijiazhuang, Hebei). PCR primers were the universal sequencing primers (P5: 5'-AATGATACGGCGACCACCGA-3'; P7: 5'-CAAGCAGAAGACGGCATACGA-3'). The PCR conditions were as follows: initial denaturation at 98℃ for 45 s, followed by 12 cycles of amplification consisting of denaturation at 98℃ for 15 s, annealing at 50℃ for 30 s, and extension at 72℃ for 30 s. A final extension was performed at 72℃ for 1 min, and the reaction was then held at 4℃ for storage. The resulting libraries containing 350—400 bp fragments were sequenced with the MGISEQ-2000 platform (MGI Tech, Shenzhen).

### Variant detection and annotation

Low-quality raw reads were trimmed with fastp v.0.20.0 with parameter: -n 10 -q 20 -u 40 (Chen et al. 2018). The resulting clean reads were aligned to the *Pst* race 134E16A + 17 + 33 + reference genome using Burrows Wheeler Alignment tool (BWA v. 0.7.17-r1188) (Li and Durbin 2009; Schwessinger et al. 2022). SNP and InDel variant detection were performed using the Genome Analysis Toolkit (GATK) pipeline (McKenna et al. 2010). Patterning processes were conducted according to the method of Xiang et al. (Xiang et al. 2023). The data were first filtered using the VariantFiltration function with the following parameters: QD < 2.0, QUAL < 30, FS > 200.0, and ReadPosRankSum < 20.0. The GATK tools HaplotypeCaller and GenotypeGVCFs were used in variant calling to create raw variant calls. SNPs were annotated by SnpEff v5.1 (Cingolani et al. 2012) using the gene annotations for *Pst* race 134E16A + 17 + 33 + primary contigs and were visualized in R package ‘ggplot2’, and SNPs in gene coding sequence (CDS) regions were annotated and statistically analyzed.

To ensure genotyping accuracy, sites with *sequencing depth (*DP) < 10 were removed. A core SNP set was derived from the remaining SNPs using a linkage disequilibrium (LD) pruning procedure in PLINK v1.9 with the parameters: ‘–indep pairwise 100 50 0.2’ (Sotiropoulos et al. 2022). For this, SNPs were removed using a window size of 100 kb, a window step size of 50 SNPs, and an *r*^*2*^ threshold of 0.2.

### Population structure, genetic diversity, and genetic differentiation analyses

SNPs were filtered using the parameters from a previous *Pst* study (Guo et al. 2022). This required removal of loci with more than two alleles, insufficient total or alternative allele read coverage (below 3), and missing data for > 25% for individual isolates. Loci with missing values or MAFs < 0.05 were removed, forming a high-quality SNP dataset (deposited to the National Genomics Data Center under project number PRJCA020812). Population structure was determined using STRUCTURE v2.3 (Pritchard et al. 2000). SNPs were filtered by linkage disequilibrium (LD) using PLINK v1.9 as described above. STRUCTURE output was processed with CLUMPP (Jakobsson and Rosenberg 2007). In the STRUCTURE analysis, individuals were categorized as admixed if their estimated ancestry proportion from any genetic group fell between 10 and 90%. This threshold was chosen to conservatively identify individuals with substantial genetic contributions from multiple sources, while excluding those that could be considered purebred (ancestry > 90%) or with only trace levels of admixture (ancestry < 10%). A phylogenetic tree was constructed with the neighbor-joining (NJ) method in the ‘ape’ R package (Alexander et al. 2009) and visualized with iTOL v6 (https://itol.embl.de/) (Purcell et al. 2007). Discriminant analysis of principal components (DAPC) was performed in the ‘adegenet’ R package (Jombart et al. 2010). Kinship heatmaps were generated based on the K-matrix using the R package ‘pheatmap’ v1.0.(Wu et al. 2021). Genetic diversity was analyzed with the ‘POPPR’ R package (Kamvar et al. 2015). Observed and expected heterozygosity (*Ho* and *He*, respectively) were estimated in PLINK v1.9 (Chen et al. 2016).

To test genetic differentiation between the six populations collected from the three regions over two years, fixation index (*Fst*) was calculated using the R package ‘hierfstat’ (Takezaki and Nei 1996). Analysis of molecular variance (AMOVA) and significance tests were performed in R to evaluate the molecular differences between samples from different years in the three regions (Excoffier et al. 1992). Nei’s genetic distance (D) (Nei 1972) was calculated using the dist.genpop function in the ‘adegenet’ R package (v2.1.10). To explicitly test for historical gene flow between populations while controlling for confounding effects of incomplete lineage sorting, we performed *D*-statistics (ABBA-BABA tests) using Dsuite software. Significant evidence of gene flow was defined by a |Z-score| exceeding 3, corresponding to a *P*-value < 0.05 (Malinsky et al. 2021). A visual migration network based on relative migrants was generated from the geneflow pattern network for different epidemic populations using the 'diversity' R package (Bai et al. 2021).

### High-altitude wind analysis

Upper-air wind field data were obtained from the European Centre for Medium-Range Weather Forecasts (https://www.ecmwf.int; downloaded from https://CDs.climate.copernicus.eu). Data for the northwest oversummering region of China were analyzed in Python using the U component and V component data with the pressure level at 700 hPa. The propagation track and diffusion range of airborne *Pst* urediniospores were simulated with HYSPLIT-4 models. HYSPLIT-4 simulations were conducted at altitudes of 1,900 m and 1,000 m, corresponding to the sampling sites in Salar Autonomous County, Xunhua, Haidong City, Qinghai province and Maiji District, Tianshui City in Gansu, respectively. For this study, the locations of the first sample collected in each season in Qinghai and Gansu were set as simulated central urediniospore source sites, with a tracking time of 5 d.

### Pipeline integration

To facilitate analysis by users unfamiliar with bioinformatics methods, a locus subsampling and SNP calling pipeline was integrated into the sequencing pipeline of MolBreeding Biotechnology Co., Ltd.

## Conclusion

This study developed and validated a novel genotyping-by-target sequencing (GBTS) chip as a versatile and efficient platform for fungal population genetics. Applied to *Puccinia striiformis* f. sp. *tritici*, the chip provided high-resolution insights into its population structure and migration dynamics in northwest oversummering region in China. Our key findings, namely the identification of three genetic groups, significant gene flow among them, and the prevalence of admixed individuals, collectively depict a highly connected and dynamic pathogen population. The GBTS chip is poised to become a core tool for smart agricultural disease surveillance. Looking forward, this platform enables several critical future directions: (1) real-time monitoring of emerging virulent races, particularly the distinct Group 3, by tracking shifts in admixture patterns and allele frequencies; (2) functional studies to link the genomic variations uncovered here to adaptive traits like virulence and fungicide resistance; and (3) expanded application of the strategy to construct similar genomic resources for other major fungal pathogens, thereby building a comprehensive framework for genomic epidemiology in crop protection.

## Supplementary Information

Supplementary Material 1: Text S1 The detailed process of sample handling from collection to sequencing.

Supplementary Material 2: Fig. S1 Single nucleotide polymorphism (SNP) distribution and classification. A Genic distribution of SNPs. Exon, Expressed region. B Distribution of SNP types among those within the coding sequence (CDS). SNV, Single nucleotide variants. Fig. S2 Assignment of isolates to genotype clusters using STRUCTURE. A Delta K (K = 2–9) distribution based on the Evanno method. The maximum Delta K value corresponds to the optimal cluster number. B Predicted isolate distribution among K clusters (represented by color). 21GS, 2021 Gansu; 21NX, 2021 Ningxia; 21QH, 2021 Qinghai; 22GS, 2022 Gansu; 22NX, 2022 Ningxia; 22QH, 2022 Qinghai. Fig. S3 Cluster distribution within the six *Puccinia striiformis* f. sp. *tritici* populations. 21GS, 2021 Gansu; 21NX, 2021 Ningxia; 21QH, 2021 Qinghai; 22GS, 2022 Gansu; 22NX, 2022 Ningxia; 22QH, 2022 Qinghai. Fig. S4 Gene flow among populations based on *D*-statistics.

Supplementary Material 3: Table S1 Genotyping-by-target sequencing (GBTS) chip sequencing results for the 225 *Puccinia striiformis* f. sp. *tritici* isolates. Table S2 Chromosomal distribution of loci included in the *Pst* 20 K chip. Table S3 Previously reported simple sequence repeat (SSR) and single nucleotide polymorphism (SNP) markers. Table S4 Multifaceted comparison between GBTS and conventional methods in wheat stripe rust population studies. Table S5 Nei's genetic distance (D, upper diagonal) and fixation index (*Fst*, lower diagonal) for each pairwise comparison of *Puccinia striiformis* f. sp. *tritici* populations. Table S6 Analysis of molecular variance among six populations of *Puccinia striiformis* f. sp. *tritici* collected from two provinces and one autonomous region in 2021 and 2022. Table S7 The *D*-statistic shows the degree of gene flow between populations. Table S8 Field records of wheat stripe rust samples collected in the northwest oversummering region during 2021 and 2022.

## Data Availability

Raw sequence reads generated in this study are deposited in the National Genomics Data Center (NGDC) (BioProject PRJCA020812). The analysis pipeline is publicly accessible at https://github.com/zengqd/PopulationGenetics/blob/main/Fungi/PstGTBSChip/Step1_1fq2vcf.sh. Numerical source data underlying all graphs in the manuscript can be found in the supplementary data file.

## Abbreviations

AMOVA: Analysis of molecular variance
CDS: Coding sequence
DAPC: Discriminant analysis of principal components
DP: Sequencing depth
DS: Disease severity
eMLG: Expected multi-locus genotype
*Fst*: Fixation index
GBTS: Genotyping-by-target sequencing
*He*: Expected heterozygosity
*Ho*: Observed heterozygosity
*I*: Shannon index
InDel: Insertion and deletion
IT: Infection type
LD: Linkage disequilibrium
MAF: Minor Allele Frequency
*Na*: Observed number of alleles
*Ne*: Effective number of alleles
*Nm*: Gene flow
*Pst*: *Puccinia striiformis* F. sp. *tritici*
QC: Quality control
SNVs: Single nucleotide variants
Ts: Transitions
TVs: Transversions
VCF: Variant Call Format
