# Supplementary material for: The development and validation of a genotyping-by-target sequencing chip for fungal population genetic analysis
Source: Stress Biol. 2026 Jan 19;6(1):7. doi: 10.1007/s44154-025-00281-2 (PMC12812796; doi:10.1007/s44154-025-00281-2)
Supplement: Supplementary file 1 — Supplementary Material 1: Text S1 The detailed process of sample handling from collection to sequencing. [file 44154_2025_281_MOESM1_ESM.docx]

**St****ep 1** **Sampling and bringing back to the lab.**

Collect single sporulating lesion-infected leaves from the field and bring them to the lab. Dry the samples at room temperature during this process.


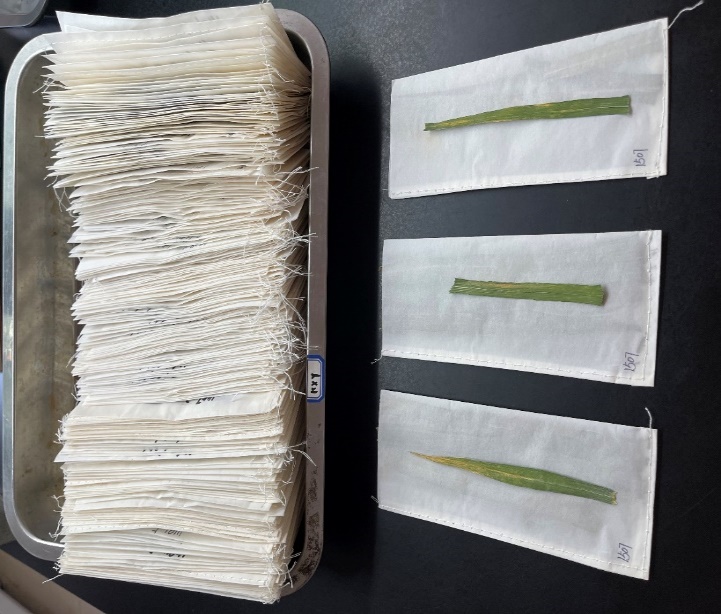


**Step 2 Experimental tools and consumables**


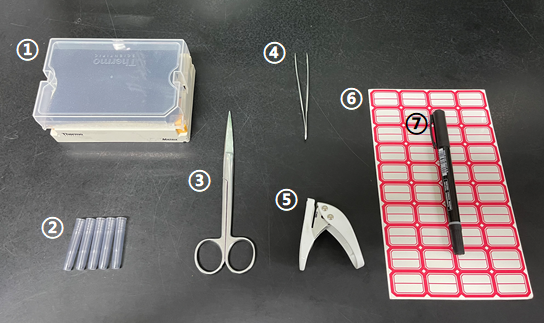


①:Sampling box; ②:Sampling tube; ③:Scissors; ④:Tweezers; ⑤: Puncher; ⑥: Label sticker; ⑦:Marker pen;

**Step 3 Punching five leaf discs from the lesions**


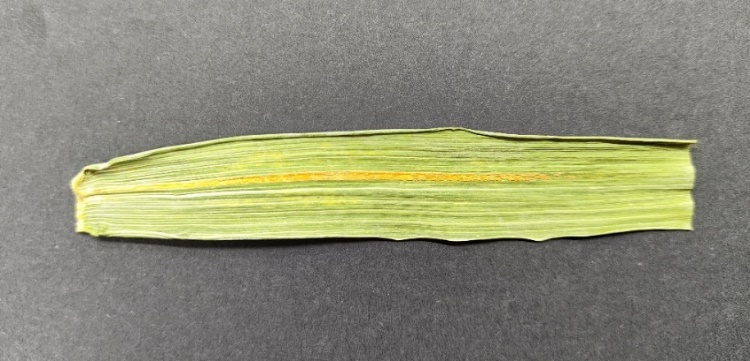

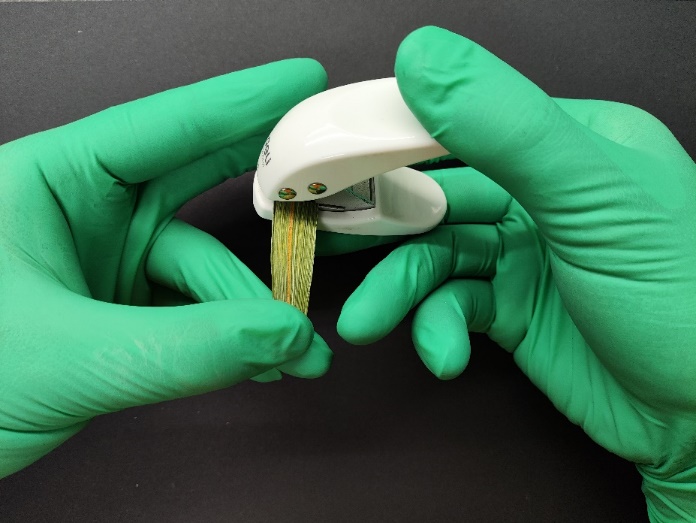

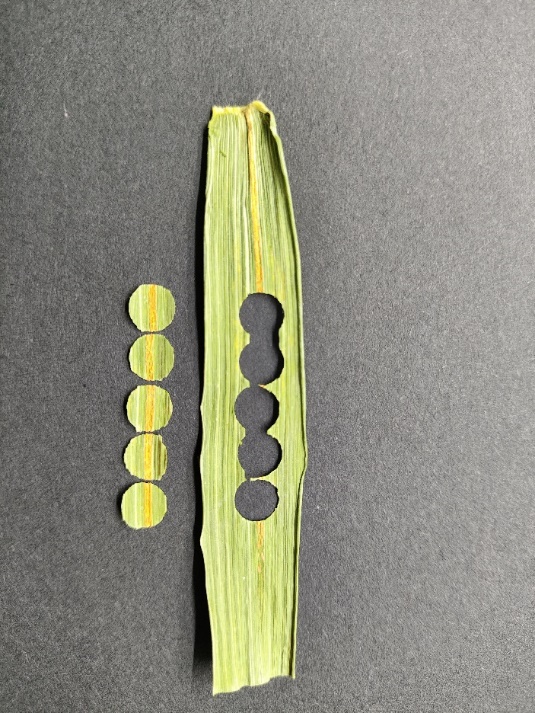


**Step 4 Place the five leaf discs from a single leaf into a single sampling tube**


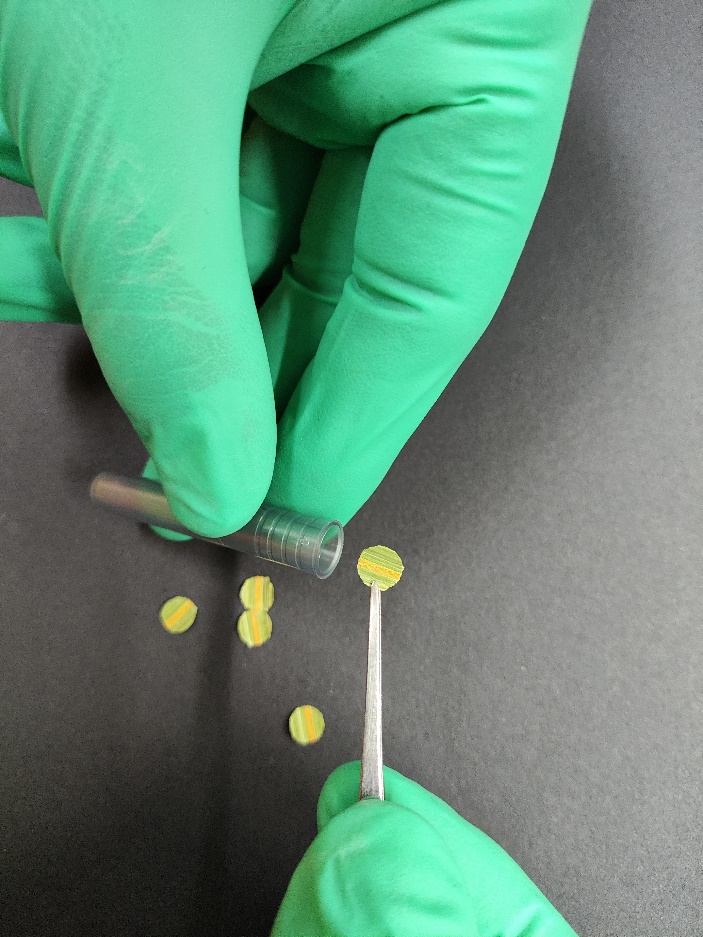

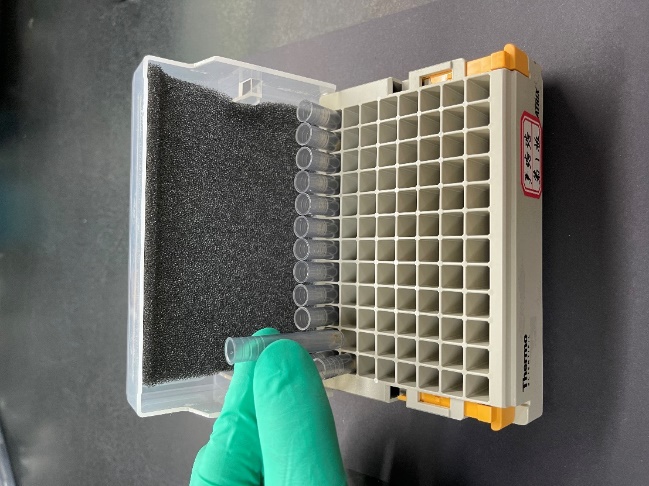


**Step 5** **Freeze dried samples (not mandatory)**

Processed leaf discs with a freeze dryer for 2 days.


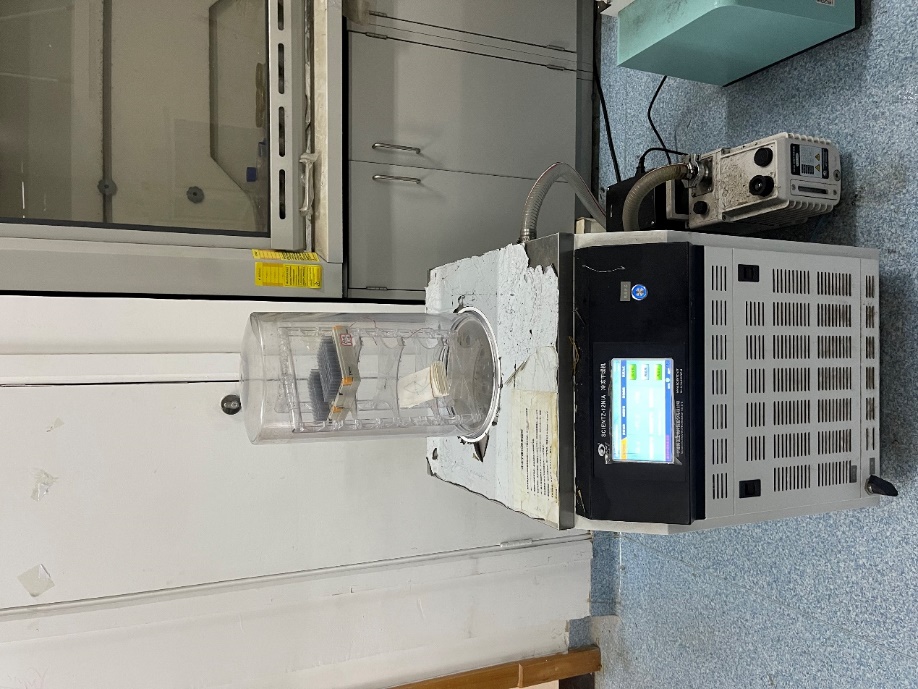


**Step 6 Sample Dispatch**

The freeze-dried or air-dried at room temperature are carefully packaged and dispatched to MolBreeding Biotechnology Company in Shijiazhuang, Hebei, for sequencing with the *Pst* 20K GBTS chip.
