# Supplementary material for: The development and validation of a genotyping-by-target sequencing chip for fungal population genetic analysis
Source: Stress Biol. 2026 Jan 19;6(1):7. doi: 10.1007/s44154-025-00281-2 (PMC12812796; doi:10.1007/s44154-025-00281-2)
Supplement: Supplementary file 2 — Supplementary Material 2: Fig. S1 Single nucleotide polymorphism (SNP) distribution and classification. A Genic distribution of SNPs. Exon, Expressed region. B Distribution of SNP types among those within the coding sequence (CDS). SNV, Single nucleotide variants. Fig. S2 Assignment of isolates to genotype clusters using STRUCTURE. A Delta K (K = 2–9) distribution based on the Evanno method. The maximum Delta K value corresponds to the optimal cluster number. B Predicted isolate distribution among K clusters (represented by color). 21GS, 2021 Gansu; 21NX, 2021 Ningxia; 21QH, 2021 Qinghai; 22GS, 2022 Gansu; 22NX, 2022 Ningxia; 22QH, 2022 Qinghai. Fig. S3 Cluster distribution within the six Puccinia striiformis f. sp. tritici populations. 21GS, 2021 Gansu; 21NX, 2021 Ningxia; 21QH, 2021 Qinghai; 22GS, 2022 Gansu; 22NX, 2022 Ningxia; 22QH, 2022 Qinghai. Fig. S4 Gene flow among populations based on D-statistics. [file 44154_2025_281_MOESM2_ESM.pptx]

## Slide 1
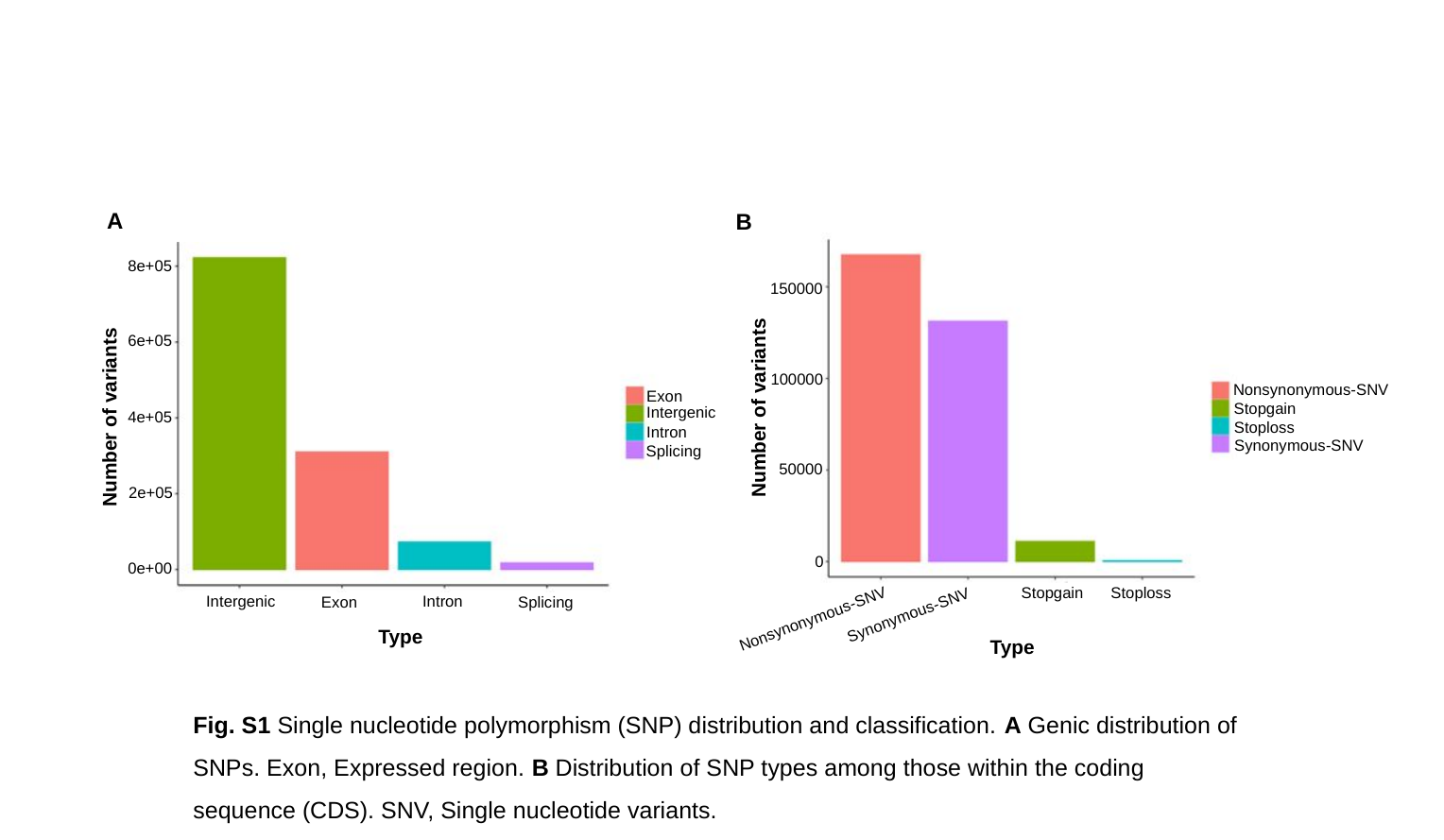

A
B
8e+05
150000
6e+05
100000
Nonsynonymous-SNV
Exon
Number of variants
Stopgain
Number of variants
Intergenic
4e+05
Stoploss
Intron
Synonymous-SNV
Splicing
50000
2e+05
0
0e+00
Stopgain
Stoploss
Intron
Intergenic
Splicing
Exon
Synonymous-SNV
Nonsynonymous-SNV
Type
Type
Fig. S1 Single nucleotide polymorphism (SNP) distribution and classification. A Genic distribution of SNPs. Exon, Expressed region. B Distribution of SNP types among those within the coding sequence (CDS). SNV, Single nucleotide variants.

## Slide 2
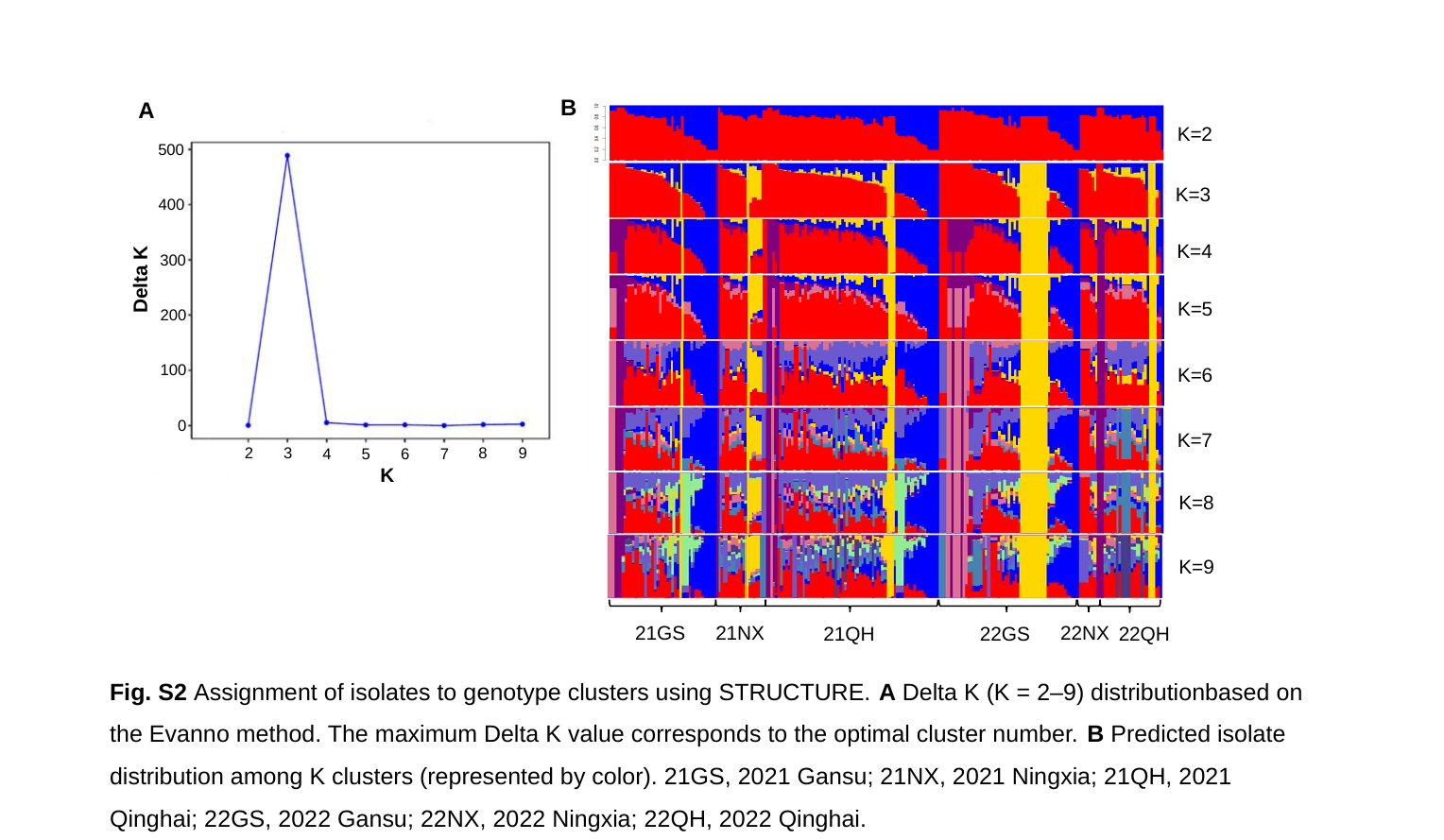

B
A
K=2
K=3
K=4
K=5
K=6
K=7
K=8
K=9
21NX
22NX
21GS
22GS
22QH
21QH
500
400
300
Delta K
200
100
0
9
2
8
3
6
7
4
5
K
Fig. S2 Assignment of isolates to genotype clusters using STRUCTURE. A Delta K (K = 2–9) distributionbased on the Evanno method. The maximum Delta K value corresponds to the optimal cluster number. B Predicted isolate distribution among K clusters (represented by color). 21GS, 2021 Gansu; 21NX, 2021 Ningxia; 21QH, 2021 Qinghai; 22GS, 2022 Gansu; 22NX, 2022 Ningxia; 22QH, 2022 Qinghai.

## Slide 3
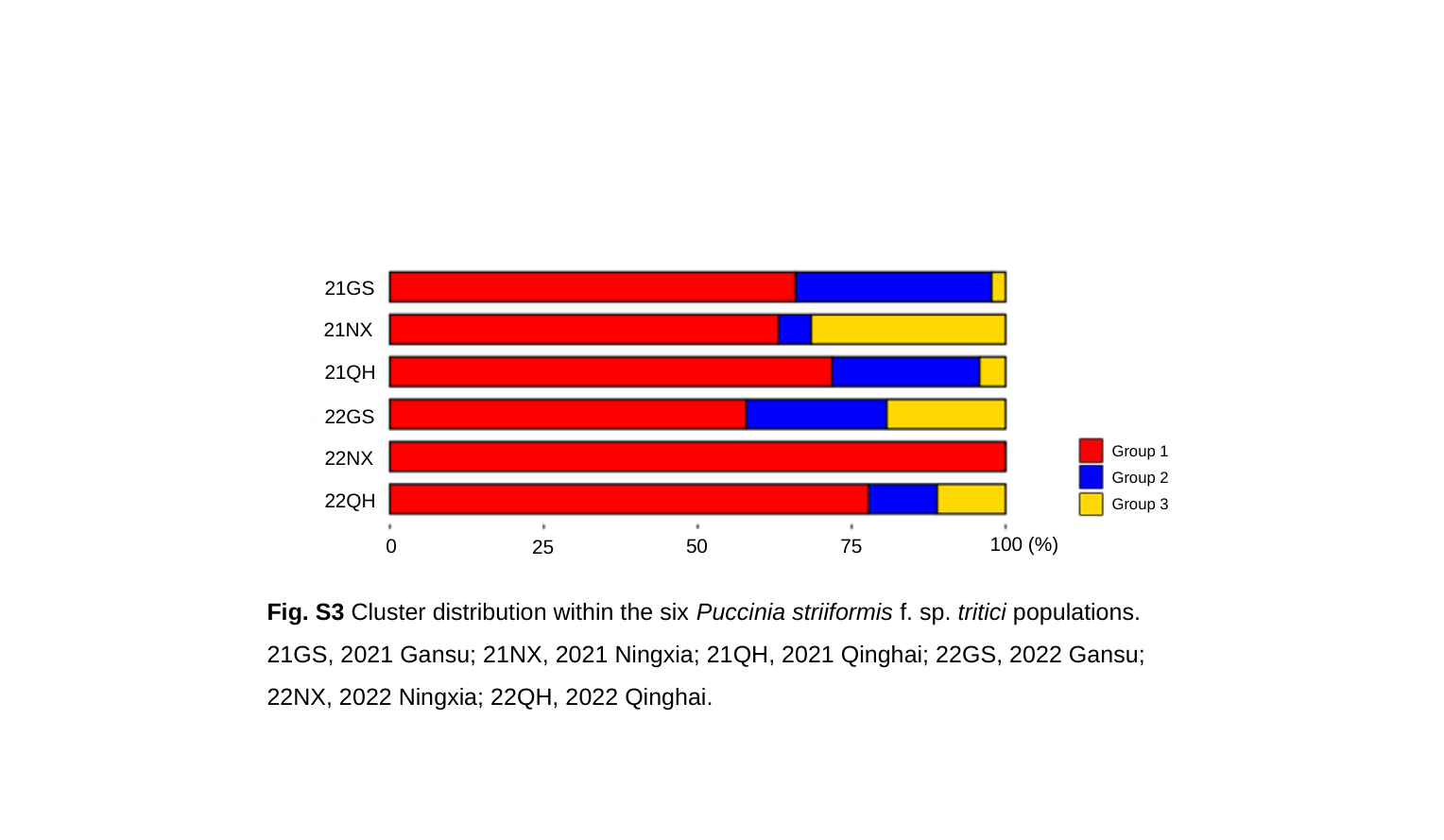

21GS
21NX
21QH
22GS
Group 1
22NX
Group 2
22QH
Group 3
100 (%)
0
50
75
25
Fig. S3 Cluster distribution within the six Puccinia striiformis f. sp. tritici populations. 21GS, 2021 Gansu; 21NX, 2021 Ningxia; 21QH, 2021 Qinghai; 22GS, 2022 Gansu; 22NX, 2022 Ningxia; 22QH, 2022 Qinghai.

## Slide 4
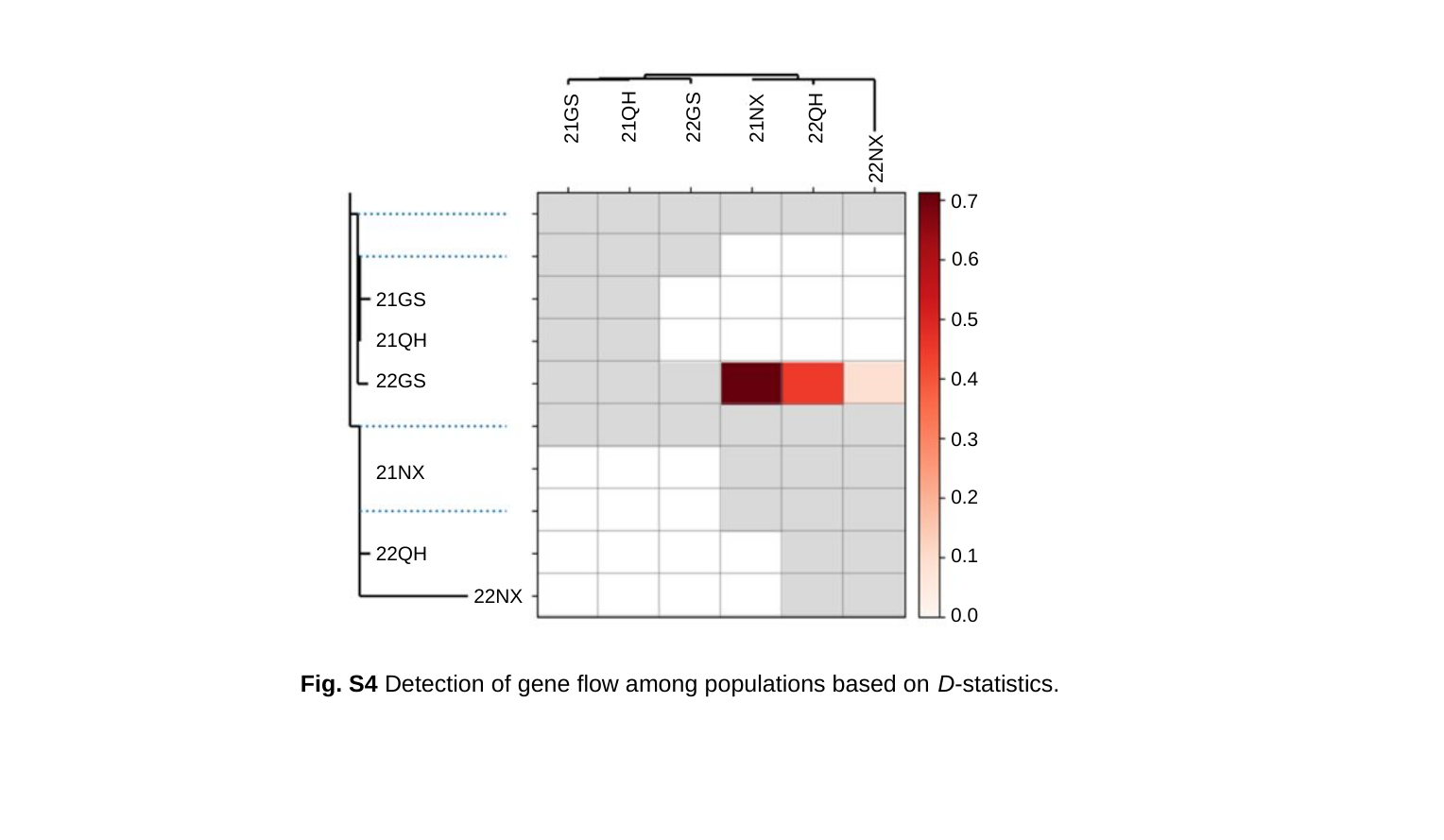

21NX
22GS
21QH
21GS
22QH
22NX
0.7
0.6
21GS
0.5
21QH
0.4
22GS
0.3
21NX
0.2
22QH
0.1
22NX
0.0
Fig. S4 Detection of gene flow among populations based on D-statistics.
